# Physical Status of Human Papillomavirus Integration in Cervical Cancer Is Associated with Treatment Outcome of the Patients Treated with Radiotherapy

**DOI:** 10.1371/journal.pone.0078995

**Published:** 2014-01-10

**Authors:** Hye-Jin Shin, Jungnam Joo, Ji Hyun Yoon, Chong Woo Yoo, Joo-Young Kim

**Affiliations:** 1 Radiation Medicine Branch, Research Institute and Hospital, National Cancer Center, Goyang, Korea; 2 Biometric Research Branch, Research Institute and Hospital, National Cancer Center, Goyang, Korea; 3 Center for Uterine Cancer, Research Institute and Hospital, National Cancer Center, Goyang, Korea; 4 Department of Microbiology & Immunology, McGill University, Montreal, Quebec, Canada; The Chinese University of Hong Kong, Hong Kong

## Abstract

Integration of human papillomavirus (HPV) DNA into the host genome is a critical aetiological event in the progression from normal cervix to intraepithelial neoplasm, and finally to invasive cervical cancer. However, there has been little work on how HPV integration status relates to treatment outcome for cervical carcinomas. In the current study, HPV E2 and E6 gene copy numbers were measured in 111 cervical cancer tissues using real-time QPCR. Integration patterns were divided into four groups: single copy-integrated with episomal components (group 1), single copy-integrated without episomal components (group 2), multicopy tandem repetition-integrated (group 3), and low HPV (group 4) groups. A relapse-predicting model was constructed using multivariable Cox proportional hazards model to classify patients into different risk groups for disease-free survival (DFS). The model was internally validated using bootstrap resampling. Oligonucleotide microarray analysis was performed to evaluate gene expression patterns in relation to the different integration groups. DFS rate was inferior in the order of the patients in group 4, group 2/3, and group 1. Multivariate analysis showed that histologic grade, clinical stage group, and integration pattern were significant prognostic factors for poor DFS. The current prognostic model accurately predicted the risk of relapse, with an area under the receiver operating characteristic curve (AUC) of 0.74 (bootstrap corrected, 0.71). In conclusion, these data suggest that HPV integration pattern is a potent prognostic factor for tailored treatment of cervical cancer.

## Introduction

Integration of human papillomavirus (HPV) DNA has been considered as a critical event in the process of cervical carcinogenesis. HPV DNA is found to be present in integrated forms in host cells with increasing frequency from normal cervical epithelium to intraepithelial neoplasias, and invasive cancer of the cervix [1], [2], [3]. However, there are reports showing that episomal HPV DNA is present in a substantial portion of invasive cervical cancers, suggesting integration is not always required for malignant progression [4], [5]. Single copy HPV integration results in disruption of the E1 or E2 open reading frame (ORF) [2], [4], . As an alternative, integration of multiple copies of HPV can occur as head-to-tail tandem repeats [6], [7]. When this occurs, only the viral copy flanking the cellular DNA is disrupted in the E1 or E2 region whilst the internal copies have intact E1 and E2 open reading frames (ORF's). Whereas loss of the E2 ORF can deregulate transcription of E6 and E7, it is possible that intact E2 ORFs, which are found with multicopy tandem HPV integrated genomes, may express and reassert transcriptional control on E6/E7. In the current study, we evaluated the prognostic significance of different HPV integration patterns for DFS from cervical cancer. Microarray analysis was then used to compare differences in total gene expression between these different integration patterns.

## Materials and Methods

### Patient characteristics

This study was approved by the Institutional Review Board of the National Cancer Center, Korea, and written informed consent was obtained from all patients. 111 consecutive patients were recruited from July 2003 to July 2008 diagnosed with HPV 16, 18, or 58 positive squamous, adeno, or adenosquamous cell carcinoma of the cervix. The clinical characteristics of these patients are presented in table 1. Diagnostic work-ups included rectosigmoidoscopy in all patients, magnetic resonance imaging (MRI) of the pelvis ± abdomen in 110 patients, and either positron emission tomography (PET) scan or PET/CT (computed tomography) scan in 93 patients. All were treated with radiotherapy which consists of external beam radiotherapy (EBRT) and high-dose-rate brachytherapy. During EBRT, concomitant weekly cisplatin 40 mg/m^2^ was given to all patients except 9 patients who had a poor performance scale. After the completion of primary treatment, patients were followed up every 3 months during the first 2 years, every 4 months during the third year, and every 6 months thereafter. Details of radiotherapy have been described previously [8].

**Table 1:** Clinicopathologic characteristics of the patients.

Characteristics	Patients (n = 111)		
	No (%)	No. of Relapse (%)	No. of No Relapse (%)
**Event**	111(100)	33 (29.7)	
**Group by Integration pattern**			
Group 1^a^	31	5	26
Group 2^b^	24	5	19
Group 3^c^	37	12	25
Group 4^d^	14	7	7
**HPV type**			
16	87	23 (26.4)	64 (73.6)
18	16	8 (50)	8 (50)
58	8	2 (25)	6 (75)
**Stage group**			
∼IIB	86	19 (22.1)	67 (77.9)
III/IVA	18	9 (50.0)	9 (50.0)
IVB	7	5 (71.4)	2 (28.6)
**Nodal status** *			
Negative	44	7 (15.9)	37 (84.1)
Positive	67	26 (38.8)	41 (61.2)
**Histologic Grade**			
well/moderate	91	24 (26.4)	67 (73.6)
Poor	19	9 (47.4)	10 (52.6)
**Histologic type**			
SCC	100 (90)	27 (27)	73 (73)
AD	9 (10)	4 (44.4)	5 (55.6)
ASC	2	2 (100)	0 (0)
**Smoking**			
Never	94 (84)	26 (27.7)	68 (72.3)
Ever	17 (16)	7 (42.2)	10 (58.8)
**Age**	median, 58;	range, 27–81	

^a^ Group 1, Single copy HPV integration with episomal components;

^b^ Group 2, Single copy HPV integration without episomal components;

^c^ Group 3, Multicopy tandem repeated integration;

^d^ Group 4, low HPV.

Abbreviations: AD/ASC, adenocarcinoma/adenosquamous carcinoma; SCC, squamous cell carcinoma.

Pelvic lymph nodes only 66, Pelvis+para-aortic lymph nodes 31.

### Real-time polymerase chain reaction of HPV E6 and E2 genes

Extraction, amplification, and genotyping of DNA were performed as previously reported [8]. Real-time PCR was carried out with ABI Prism 7900HT System and TaqMan Universal PCR Master Mix (PE Applied Biosystems, Perkin-Elmer, Foster City, CA). The primers and probes were designed for specific amplification of the E2 hinge regions which are known to be disrupted most frequently during the process of viral integration [2]. The sequence information of each primers used are available in Table S1. Fifty nanograms of target DNA from each biopsy specimen were used per reaction and standard curves were produced by amplification of a dilution series of 10^6^ -10^3^ copies of a clone of HPV DNA in pBR322. At least three no-template control reaction mixtures were included in each trial and all experiments were performed in triplicate.

### Classification of the cervical tumors with physical status of HPV genomes

On the basis of the relative copy numbers of E2 and E6, patients were classified into 4 groups; Single copy-integrated with episomal components (group 1) and without episomal components (group 2), multicopy tandem repetition-integrated (group 3), and low HPV (group 4) groups. Group 1 tumors still contain episomal HPV with amplification of both E6 and E2 although levels of E2 are reduced. Group 2 tumors have no E2 amplification. Group 3 tumors have equal levels of E2 and E6 which indicates multicopy tandem repeated HPV integration. Group 4 tumors have no E2 and E6 amplifications. These four groups were eventually re-classified into three groups based on their different clinical outcomes (group 1 vs. group 2/3 vs. group 4).

### Microarray analysis

Microarray analysis was performed for 39 patients from whom freshly frozen tumor samples could be obtained. Only the patients with HPV16 infection were included in the microarray experiment to avoid heterogeneity caused by different HPV genotypes. As a result, 14, 9, 12, and 4 samples from integration groups1 to 4, respectively, were analyzed using whole human gene expression microarray 44K v.1.0 (Agilent Technologies, Inc., Palo Alto, CA). All numerical data were normalized with per-spot and per-chip normalization (intensity-dependent normalization), and per-gene normalization (to median) using GeneSpring 7.3.1. Rank-based linear trend analysis was used to test the linear trend in gene expressions between different integration patterns ordered by clinical outcomes. Permutation method with 250,000 re-sampling was performed to evaluate the corresponding *p*-value. The expression levels in log2 scale were used in the analysis, and the false discovery rate (FDR) [9] was controlled at 0.05. Microarray data have been deposited in the Gene Expression Omnibus (http://www.ncbi.nlm.nih.gov/geo/) and are accessible through Gene Expression Omnibus series number GSE49288.

### Statistical analysis

Demographic and clinical characteristics are presented as counts and percentages for categorical variables, and median and range for continuous variables. The distributional differences between groups were assessed using the Pearson Chi-square test or Fisher's exact test. Spearman's rank correlation was obtained to measure the association between HPV viral load and copy numbers. The differences in copy numbers between groups were tested using Kruskal-Wallis test. The multivariable Cox proportional hazards model was employed to predict the DFS after adjusting the effects of other potential confounding factors such as age, stage group, nodal status, histologic grade, and histologic type. The final model was chosen based on the result of univariable analysis as well as consideration of the clinical or biological importance of the variables. All statistical analysis were performed using SAS version 9.1 (Cary, NC) and R statistical software, version 2.12. All reported *p*-values are two-sided.

### Validation of the prediction model using bootstrap resampling

The discrimination ability of the multivariable Cox model for predicting 2 year disease free survival was assessed using the C statistic, a concordance measure analogous to area under the receiver operating characteristic (ROC) curve. Calibration of the model was evaluated using Hosmer-Lemeshow type chi-square test by comparing the difference between the observed and predicted 2 year survival rates by the developed model for patients in each quintile. Based on the predicted survival rate, three groups were formed by dividing the patients into tertiles. The difference in survival distributions of these three groups were compared using the log-rank test. The predictive model was validated using 1000 bootstrap samples as an internal validation to correct the upward bias in estimating C statistic due to re-substituting the data.

## Results

### E6 and E2 gene copy numbers and integration pattern

**Figure 1:**
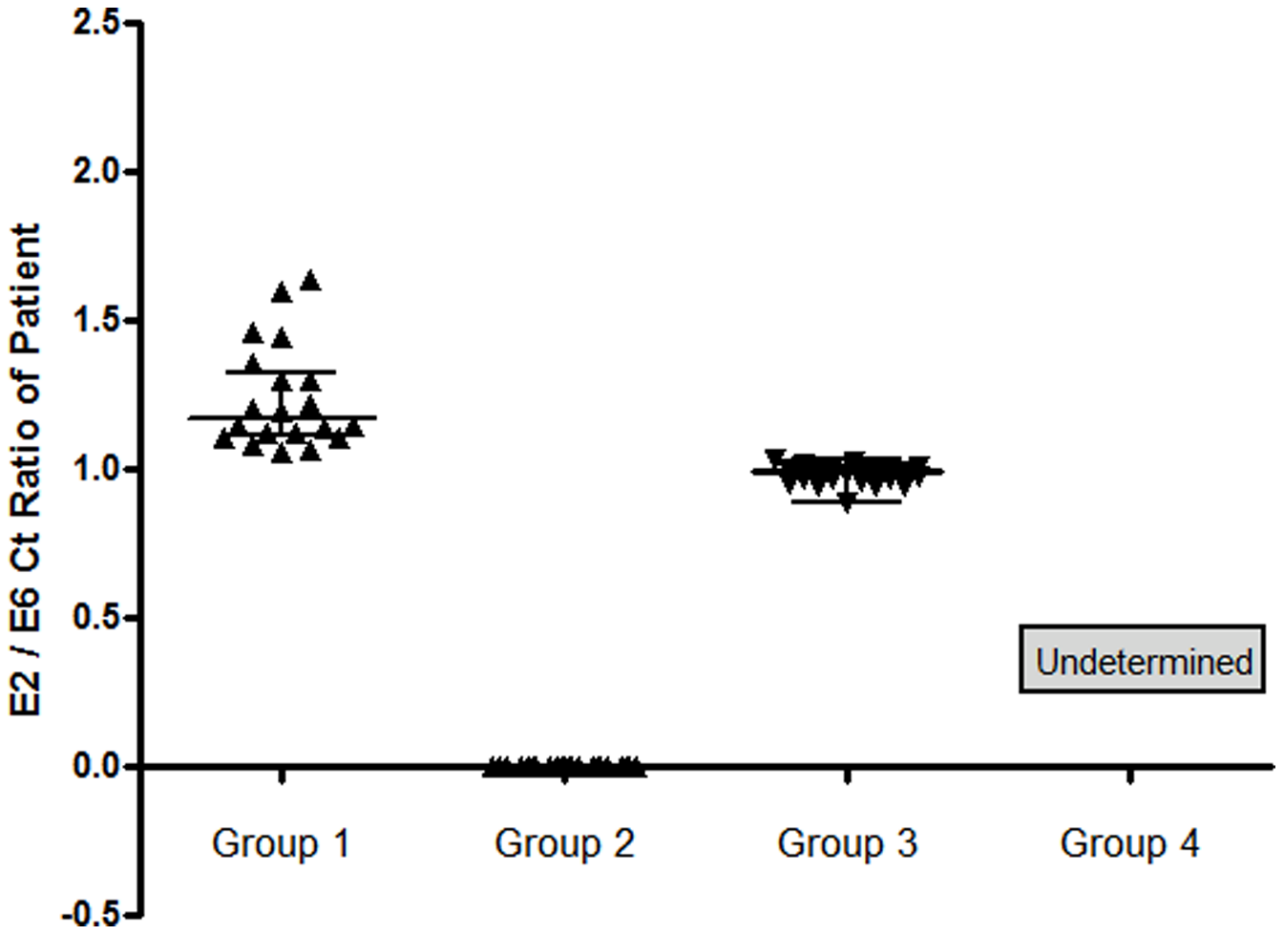
Each Ct value of HPV E2 and E6 gene copies was measured in 111 cervical cancer patients using real-time QPCR. The E2/E6 Ct ratios of the patients in each group are depicted as dots on the scatter plot. The horizontal bars represent the median value with interquartile range.

Classification of the patients based on the relative amount of E2 and E6 copy numbers resulted in 31, 24, 37, and 14 patients in groups 1 to 4, respectively. The threshold cycle (Ct) ratios of E2/E6 from the patients in each group are shown by a scatter plot (Fig. 1). 5 patients had unclassifiable patterns. E6 and E2 copy numbers showed a positive correlation in group3 (r = 0.83, *p*<0.0001) and with group1 (r = 0.43, *p = 0.02*) but not in group2 (r = 0.008, *p* = 0.97) nor in group 4 (r = 0.26, *p = 0.37*).

### Univariable and multivariable analysis of treatment outcome

**Figure 2:**
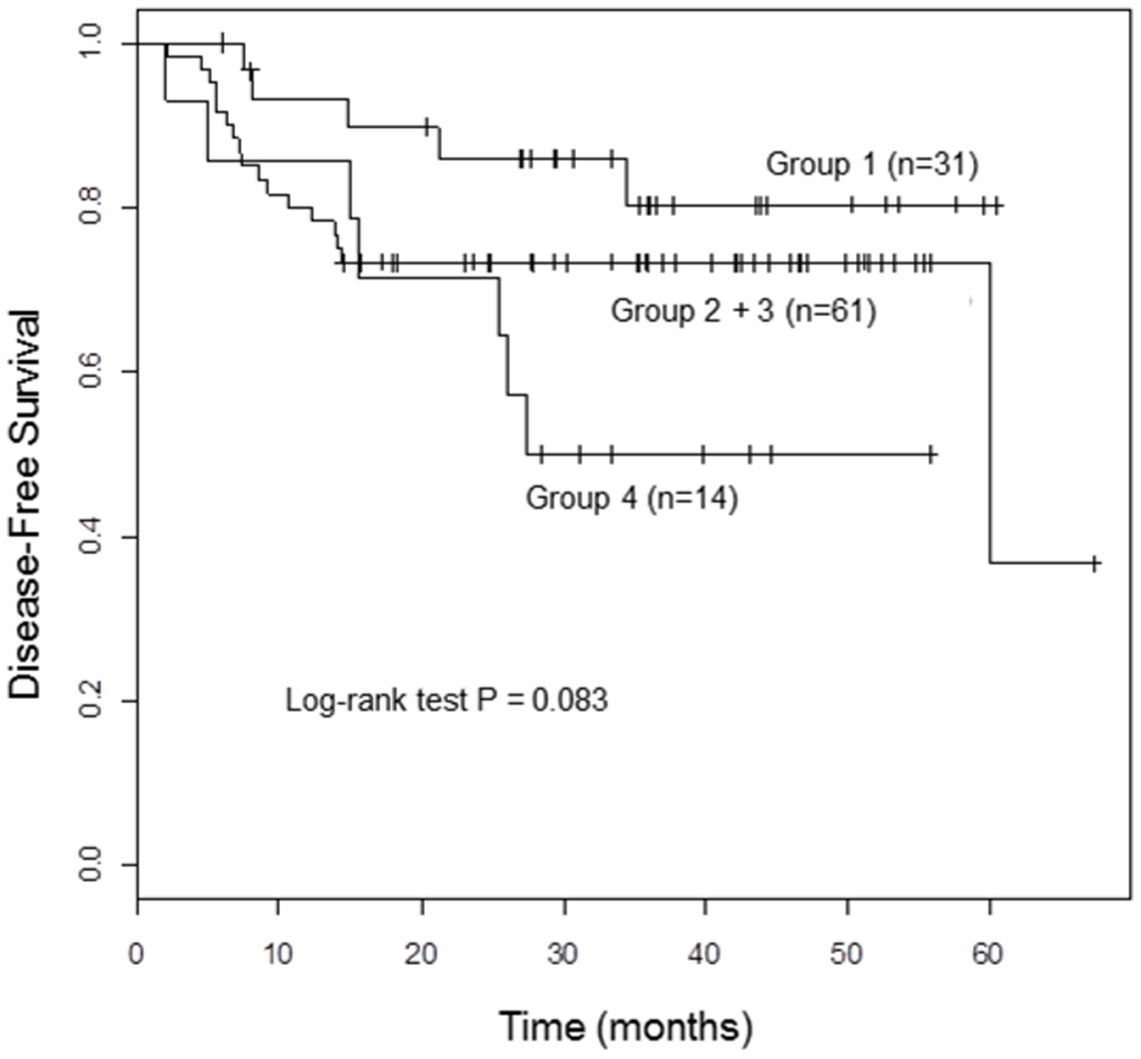
Disease-free survival rates of the patients by physical status of HPV DNA in cervical cancer. Group 1; Single copy HPV integration with episomal components, Group 2; Single copy HPV integration without episomal components, Group 3; Multicopy tandem repeated integration, Group 4; low HPV.

During the follow-up, 33 patients (29.7%) developed disease progression including 25 distant metastases or 10 local recurrences. Amongst these patients, three had both. Thirteen patients died and the cause of death of 2 patients among them was cardiovascular disease and unknown, respectively. They were censored on the date of death. The patients were followed up for median 29 months (range, 2–67 months). The median period of follow-up for the patients without recurrence was 36 months (range, 6–67 months). When the four groups were re-classified into three groups, the DFS rate of the patients in group 1 was the highest, followed by groups 2/3 and group 4 in a descending order. The hazard ratios (HR) and confidence interval was 1.90, Confidence Interval (CI) 0.70–5.15, *p* = 0.207 for group 2/3 against group 1, HR 3.52, CI 1.11–11.14, *p* = 0. 032 for group 4 against group 1, respectively (*p* = 0.083 by log-rank test, Fig. 2 and Table 2) on univariable analysis. Other significant prognostic parameters for DFS included stage group, nodal metastasis, histologic grade, histologic type, and smoking by univariable analysis. In multivariable analysis, integration pattern (overall *p* = 0.014; for group 2/3 vs. group 1, *p* = 0.03, HR 3.15, CI 1.12–8.9; for group 4 vs. group 1, *p* = 0.004, HR 6.78, CI 1.86–24.79), histologic grade, clinical stage group, and age remained significant (Table 2).

**Table 2:** Univariable and multivariable analysis for disease-free survival with clinicopathologic prognostic factors.

	Disease Free Survival			
	Univariable hazard ratio (95% CI)	*p*-value	Multivariable hazard ratio (95% CI)	*p*-value
**Integration Pattern**		(0.098)		(0.014)
Group1	1		1	
Group2+3	1.90 (0.70, 5.15)	0.207	3.15 (1.12,8.90)	0.03
Group4	3.52 (1.11,11.14)	0.032	6.78 (1.86,24.79)	0.004
**HPV type**				
16	1			
18	1.89 (0.84, 4.23)	0.124		
58	0.92 (0.22, 3.92)	0.914		
**Stage group**				(0.005)
∼IIB	1		1	
III/IVA	2.79 (1.25, 6.21)	0.012	3.83 (1.56, 9.39)	0.003
IVB	4.33 (1.59, 11.78)	0.004	3.62 (1.15,11.44)	0.028
**Nodal state**				
Negative	1		1	
Positive	2.75 (1.19, 6.36)	0.018	1.14 (0.40, 3.19)	0.81
**Histologic Grade**				
well/moderate	1		1	
Poor	1.97 (0.91, 4.26)	0.083	2.53 (1.01, 6.34)	0.047
**Histologic type**				
SCC	1			
AD/ASC	2.301 (0.94, 5.65)	0.069		
**Smoking**				
Never	1			
Ever	1.65 (0.71, 3.81)	0.244		
**Age** *	0.85 (0.75,0.97)	0.014	0.93 (0.69, 0.98)	0.034

Abbreviations: AD/ASC, adenocarcinoma/adenosquamous carcinoma; SCC, squamous cell carcinoma.

5-year increase in age.

### The performance of outcome-predicting models and internal validation

**Figure 3:**
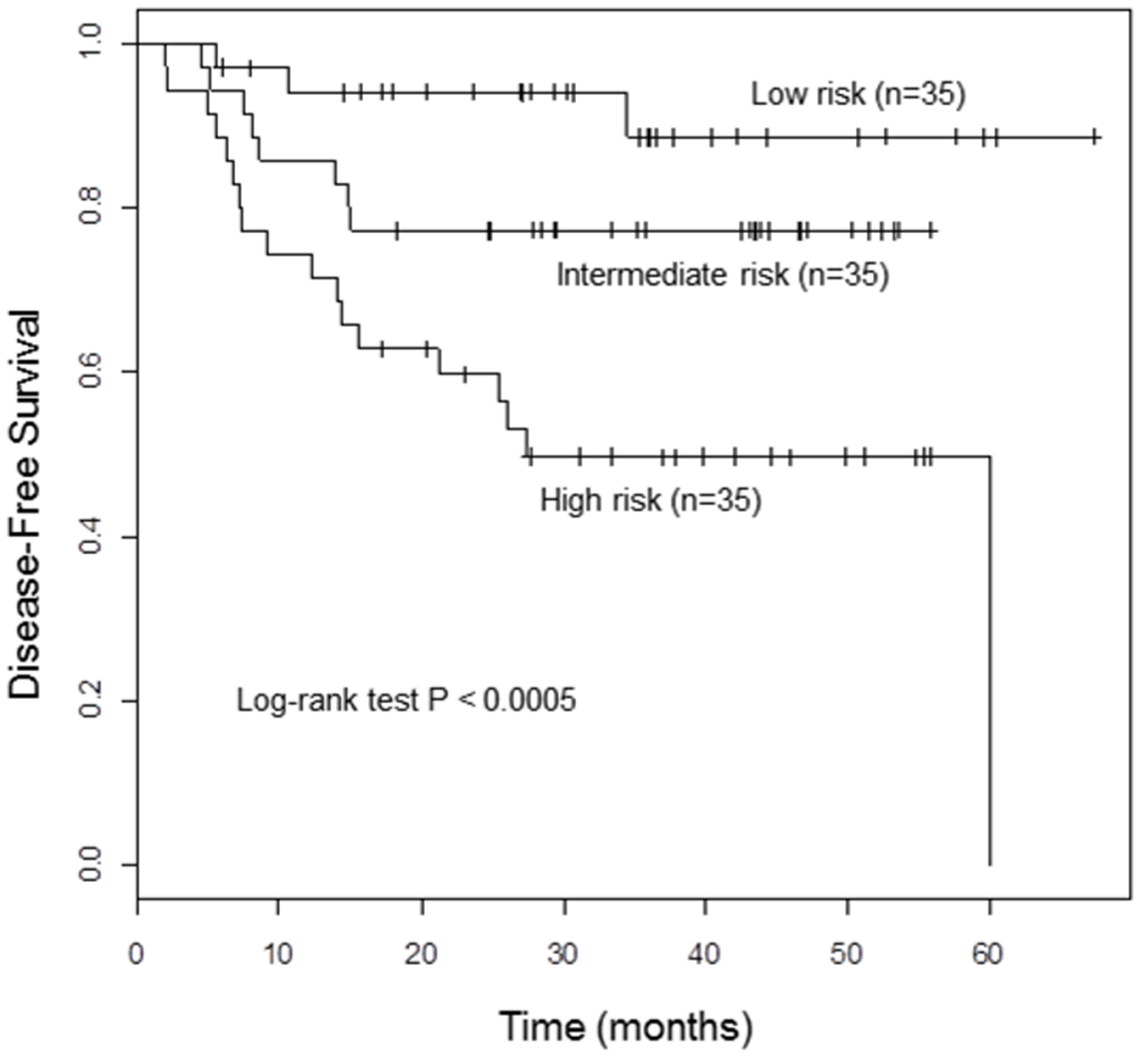
Disease-free survival of the patients by three risk groups according to the predicted risk probability.

The C-statistic of the model to predict 2 year survival was 74% (95% CI, 65%–83%) and the bootstrap validated C-statistic was 71%. The Hosmer-Lemeshow type Chi-square test statistic was 9.02 with the corresponding *p* = 0.435 which indicates a good fit. The log-rank test resulted in a significant difference between survival distributions of the three groups of patients defined by the predicted survival rate (*p*<0.0005) (Fig. 3 and Table 3).

**Table 3:** Hazard ratios (HR) of disease-free survival for three groups according to predicted risk probability.

Risk Group	Estimated 1 year survival	Estimated 2 year survival	No. of Patients (%)	Event (%)	Disease Free Survival		
					HR	95% CI	*p*-value
**Low**	0.955	0.929	35 (33.3)	3 (10)	1		0.002
**Intermediate**	0.874	0.805	35 (33.3)	8 (28)	2.94	(0.78,11.18)	0.11
**High**	0.712	0.578	35 (33.3)	18 (62)	7.42	(2.18,25.23)	0.001

### Microarray analysis of the 39 cervical cancer samples

**Figure 4:**
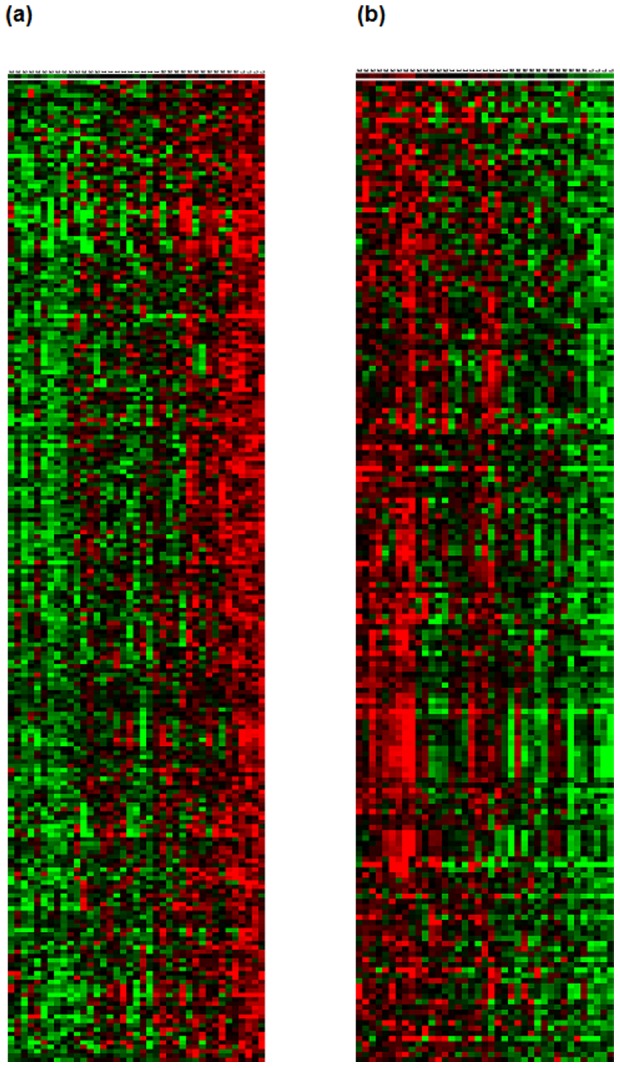
Heat map of the genes with significant linear trend from group1 to group4, increasing (a) and decreasing expression (b).

654 genes were selected as showing significantly different expressions among the integration groups classified by the physical status of HPV DNA (group 1 vs. group 2/3 vs. group4). Because the patients' survival rates tend to decrease from group1 to 4, genes that exhibited a correlative trend in their expression levels were examined. As a result, the mean expressions of 429 genes among them were shown to be in gradual increase (241) or decrease (188) in the order of group1 to 4 (Fig. 4). After a stringent selection based on the family wise error rate (Bonferroni correction) less than 5%, transmembrane modulators of cell adhesion such as claudin, occludin, and syndecan remained as genes that show significant decreases from group 1 to 4. Regulators of small GTPase-mediated signal transduction such as cell division control protein 42 (CDC42) effector proteins, Rho GTPase activating proteins, and p21 (CDC42/Rac)-activated kinase 2 (PAK2) also significantly decreased. These genes are known to play an important role in reorganization of actin stress fibers, adhesion of cell to cell, cell to extracellular matrix. Among the genes with increasing trend of expression from group1 to 4 were transcription regulators, such as MYC-associated factor X (MAX) and tumor necrosis factor (TNF). Also, there were molecules associated with chromatin remodeling such as AT-rich interactive domain 1A (SWI-like), AT-rich interactive domain 4B (RBP1-like, BRCAA1), and chromodomain helicase DNA binding protein. Biological functions of these genes were examined using the DAVID Bioinformatics Resources version 6.7 (http://david.abcc.ncifcrf.gov/). Detailed information of the genes and *p*-value of the linear trend are listed in Table S2. Gene ontology analysis using this tool indicates that the level of expression of genes related to keratinocyte and T-lymphocyte differentiation was very low in group 4. Especially, retinoid X receptor alpha (NM_002957) was significantly decreased in group 4 while it was most highly expressed in group 1 (data not shown). This supports the observation of lack of epithelial differentiation in tumors with low HPV DNA.

## Discussion

In the current study, the prognostic value of the physical status of HPV was examined in locally advanced cervical tumors. Our study reveals that cervical tumors with episomal components have superior post-radiotherapy survival when compared to those with purely integrated HPV. It was also found that the prognosis of cervical cancer is significantly decreased in tumors with undetectable level of HPV DNA.

It has been previously shown that invasive tumors are not always composed of cells with 100% integrated HPV DNA. There are approximately 17∼20% of the patients with invasive cancer with mixed episomal and integrated forms of HPV [4], [5] or even episomal forms only in 7% of patients [4]. Furthermore, several studies have demonstrated that the physical status of HPV in cervical cancer is associated with treatment outcomes [4], [10], [11]. Viral integration patterns have been evaluated by southern blotting of E1/E2 site [10] or by in situ viral DNA signal amplification method [11] or both of these methods [4].

Relative amounts of E2 and E6 were used in this study and, surprisingly, it was found that one-third (37/111) of samples had multicopy HPV integration. The presence of multicopy HPV tandem repeat genomes in tumors is known to be produced by the rolling circle replication method of HPV [12]. While the prognosis of the patients with both single copy HPV integration without episomal components and multicopy tandem repeated integration was poor in our study, the biological significance of this observation needs to be clarified.

Despite HPV genotyping and rigorous real-time PCR, there were 14 patients with undetectable HPV E2 and E6 in their tumors who showed the worst survival rate among the 4 integration groups. It is possible that tumors with no HPV DNA may represent those with more characteristics of cancer stem cells which lack epithelial differentiation where viral replication is restricted with abortive HPV replication [13]. Consistent with this hypothesis, the microarray analysis showed low expression of genes related to keratinocyte and T-lymphocyte differentiation. Moreover, based on this data, it is also assumed that intergroup differences in chromatin remodeling may induce the differential expression of the various genes. Especially, the molecules associated with Rho-GTPase signaling may contribute to the development into metastatic phenotype through weakening of cell adhesion and morphologic alteration like EMT (epithelial-mesenchymal transition) in the tumors with poor survival rates as suggested in our previous study [14]. More studies must be done to prove this idea.

The current data are consistent with our previous work which described high viral load (HC2test; Digene Corp) was strongly associated with a good prognosis while the patients with lower viral load fared much worse after radiotherapy [8]. In summary, we have determined HPV integration patterns using a novel quantitative PCR method of analyzing the stoichiometry of HPV E2 and E6 and determined their relationship to post-radiation clinical outcome. Integration patterns were thus found to be a strong prognostic factor showing a good correlation with other known prognostic factors such as stage, nodal status, histologic type and differentiation status. Further study using larger sample numbers and additional methods of examination of HPV integration patterns in cervical cancer will be needed to confirm these findings.

## Supporting Information

Table S1 **Primer and probe sequences for E2 and E6 gene real-time PCR.**
(DOC)

Table S2 **Genes which showed increasing (a) and decreasing (b) linear trend from group1 to group4; The name, function, fold change (FC) of the genes and p-value of the linear trend are shown under gene ontology classification.**
(DOC)
