# Correction: Physical Status of Human Papillomavirus Integration in Cervical Cancer Is Associated with Treatment Outcome of the Patients Treated with Radiotherapy

**DOI:** 10.1371/journal.pone.0335489

**Published:** 2025-10-29

**Authors:** Hye-Jin Shin, Jungnam Joo, Ji Hyun Yoon, Chong Woo Yoo, Joo-Young Kim

After publication of this article [1], it was raised that there is an error in the Author Contributions. The corresponding author stated that the third author, Ji Hyun Yoon, contributed to the interpretation of the data regarding whether the relative amount of E2 and E6 gene measured by PCR could be used in determining the integration pattern of the HPV genome.

The Author Contributions statement is updated to: Conceived and designed the experiments: JYK HJS. Performed the experiments: HJS CWY. Analyzed the data: JJ JYK HJS JHY. Contributed reagents/materials/analysis tools: JJ JYK HJS. Wrote the paper: JYK JHY JJ HJS.

The first author stated that the data underlying this article [1] are no longer available.
