# Supplementary material for: Physical Status of Human Papillomavirus Integration in Cervical Cancer Is Associated with Treatment Outcome of the Patients Treated with Radiotherapy
Source: PLoS One. 2014 Jan 10;9(1):e78995. doi: 10.1371/journal.pone.0078995 (PMC3888442; doi:10.1371/journal.pone.0078995)
Supplement: Table S1 — Primer and probe sequences for E2 and E6 gene real-time PCR. (DOC) [file pone.0078995.s001.doc]

| **Name** | **Forward primer** | **Reverse primer** | **Probe** |
| --- | --- | --- | --- |
| **HPV 16-E2** | 5’ AAC GAA GTA TCC TCT CCT GAA ATT ATT AG 3’ | 5’ CCA AGG CGA CGG CTT TG 3’ | VIC-5’ CAC CCC GCC GCG ACC CAT A 3’ – MGB/NFQ |
| **HPV 16-E6** | 5’ ACC GGT CGA TGT ATG TCT TGT TG 3’ | 5’ GAT CAG TTG TCT CTG GTT GCA AAT C 3’ | FAM-5’ TGC ATG GAG ATA CAC CTA CAT TGC ATG AAT ATA 3’ – TAMRA |
| **HPV 18-E2** | 5’ GGT GGT GCC AGC CTA TAA CAT T 3’ | 5’ CCA TAG TTC CTC GCA TGT GTC TT 3’ | VIC-5’ AAA AGT AAA GCA CAT AAA GCT ATT GAA CTG CAA ATG GC 3’ – MGB/NFQ |
| **HPV 18-E6** | 5’ AAT ACT ATG GCG CGC TTT GAG 3’ | 5’ TTC AAA TAC CTC TGT AAG TTC CAA TAC TG 3’ | FAM-5’ TAC AAG CTA CCT GAT CTG TGC ACG GAA CTG 3’ – TAMRA |
| **HPV 58-E2** | 5’ GAG GCC ACC AAC AAC GAA AG 3’ | 5’ GTC CAC GGC GCA GTC TGT ATA 3’ | VIC-5’ AAG CGA CGA CGA CTC GAT TTA CCA GAC TC 3’ – MGB/NFQ |
| **HPV 58-E6** | 5’ TGA CAG CTC AGA CGA GGA TGA A 3’ | 5’ CAC AAG TGT AAC AAC AAG TTA CAA TGT AGT 3’ | FAM-5’ ACA AGA ACA ACC GGC CAC AGC TAA TT 3’ – TAMRA |
