# Supplementary material for: Physical Status of Human Papillomavirus Integration in Cervical Cancer Is Associated with Treatment Outcome of the Patients Treated with Radiotherapy
Source: PLoS One. 2014 Jan 10;9(1):e78995. doi: 10.1371/journal.pone.0078995 (PMC3888442; doi:10.1371/journal.pone.0078995)
Supplement: Table S2 — Genes which showed increasing (a) and decreasing (b) linear trend from group1 to group4; The name, function, fold change (FC) of the genes and p-value of the linear trend are shown under gene ontology classification. (DOC) [file pone.0078995.s002.doc]

Table S2 (a)

| Gene Ontology | Gene Title | Genbank ID | Gene Symbol | FC Group1/4 | FC Group2/4 | FC Group3/4 | qvalues |
| --- | --- | --- | --- | --- | --- | --- | --- |
| **Regulation of transcription** | B double prime 1, subunit of RNA polymerase III transcription initiation factor IIIB | NM_018429 | BDP1 | 0.63 | 0.80 | 0.97 | 0.02 |
|  | EP300 interacting inhibitor of differentiation 1 | NM_014335 | EID1 | 0.52 | 0.56 | 0.61 | 0.05 |
|  | GA binding protein transcription factor, alpha subunit 60kDa | NM_002040 | GABPA | 0.62 | 0.70 | 0.92 | 0.04 |
|  | HMG-box transcription factor 1 | NM_012257 | HBP1 | 0.66 | 0.67 | 0.89 | 0.04 |
|  | MYC associated factor X | NM_145113 | MAX | 0.65 | 0.80 | 0.83 | 0.05 |
|  | jun D proto-oncogene | X56681 | JUND | 0.74 | 0.74 | 0.83 | 0.03 |
|  | similar to Bcl-2-associated transcription factor 1 (Btf); BCL2-associated transcription factor 1 | NM_014739 | BCLAF1 | 0.70 | 0.83 | 0.85 | 0.05 |
|  | tumor necrosis factor (TNF superfamily, member 2) | NM_000594 | TNF | 0.46 | 0.67 | 0.78 | 0.03 |
|  | v-Ki-ras2 Kirsten rat sarcoma viral oncogene homolog | NM_033360 | KRAS | 0.59 | 0.77 | 0.82 | 0.04 |
|  |  |  |  |  |  |  |  |
| **RNA processing** | DEAD (Asp-Glu-Ala-Asp) box polypeptide 17 | NM_006386 | DDX17 | 0.50 | 0.57 | 0.66 | 0.02 |
|  | RNA (guanine-7-) methyltransferase | NM_003799 | RNMT | 0.73 | 0.77 | 0.88 | 0.05 |
|  | RNA binding motif protein 16 | NM_014892 | RBM16 | 0.64 | 0.72 | 0.84 | 0.03 |
|  | Wilms tumor 1 associated protein | NM_004906 | WTAP | 0.66 | 0.77 | 0.93 | 0.04 |
| **Chromatin remodeling** | AT rich interactive domain 1A (SWI-like) | NM_006015 | ARID1A | 0.60 | 0.69 | 0.83 | 0.02 |
|  | AT rich interactive domain 4B (RBP1-like) | NM_016374 | ARID4B | 0.52 | 0.56 | 0.85 | 0.01 |
|  | chromodomain helicase DNA binding protein 6 | NM_032221 | CHD6 | 0.44 | 0.51 | 0.61 | 0.03 |
|  | chromodomain helicase DNA binding protein 9 | NM_025134 | CHD9 | 0.54 | 0.62 | 0.74 | 0.03 |
|  | lysine (K)-specific demethylase 6A | NM_021140 | UTX | 0.64 | 0.70 | 0.86 | 0.05 |
|  | telomeric repeat binding factor 2, interacting protein | NM_018975 | TERF2IP | 0.57 | 0.60 | 0.72 | 0.04 |

* Highly significantly increasing gene from group1 to group4 on stringent selection of the genes based on the family wise error rate less than 5%

Table S2 (b)

| Gene Ontology | Gene Title | Genbank ID | Gene  Symbol | FC Group1/4 | FC  Group2/4 | FC Group3/4 | qvalues |
| --- | --- | --- | --- | --- | --- | --- | --- |
| **Small GTPase mediated signal transduction** | CDC42 small effector 2 | NM_020240 | CDC42SE2 | 1.25 | 1.23 | 1.02 | 0.05 |
|  | CDC42 effector protein (Rho GTPase binding) 5 | NM_145057 | CDC42EP5 | 1.58 | 1.57 | 1.05 | 0.04 |
|  | Rho GTPase activating protein 27 | NM_199282 | ARHGAP27 | 1.41 | 1.40 | 1.04 | 0.04 |
|  | p21 protein (Cdc42/Rac)-activated kinase 2 | NM_002577 | PAK2 | 2.78 | 2.31 | 1.74 | 0.00 |
| **Actin cytoskeleton organization** | cofilin 1 (non-muscle) | NM_005507 | CFL1 | 1.80 | 1.77 | 1.43 | 0.03 |
| **& cell adhesion** | fascin homolog 1, actin-bundling protein (Strongylocentrotus purpuratus) | NM_003088 | FSCN1 | 3.79 | 3.18 | 1.61 | 0.04 |
|  | thyroid hormone receptor interactor 10 | NM_004240 | TRIP10 | 2.22 | 2.17 | 1.38 | 0.05 |
|  | Claudin 4 | NM_001305 | CLDN4 | 9.71 | 2.71 | 1.60 | 0.00 |
|  | Syndecan 4 | NM_002999 | SDC4 | 2.83 | 1.65 | 1.31 | 0.04 |
|  | Occludin | NM_002538 | OCLN | 3.03 | 2.30 | 1.91 | 0.03 |
|  | Interferon induced transmembrane protein 5 | NM_001025295 | IFITM5 | 1.37 | 1.34 | 1.07 | 0.05 |
| **Negative regulation of cell proliferation** | Prohibitin | NM_002634 | PHB | 1.76 | 1.64 | 1.40 | 0.02 |
|  | Growth arrest-specific 2 like 3 | BX649059 | GAS2L3 | 1.32 | 1.29 | 1.04 | 0.05 |
|  | SMYD family member 5 | NM_006062 | SMYD5 | 1.62 | 1.52 | 1.15 | 0.02 |
|  | retinoid X receptor, alpha | NM_002957 | RXRA | 1.96 | 1.52 | 1.43 | 0.05 |

* Highly significantly decreasing gene from group1 to group4 on stringent selection of the genes based on the family wise error rate less than 5%
